# Dynamic alterations in early intestinal development, microbiota and metabolome induced by *in ovo* feeding of *L*-arginine in a layer chick model

**DOI:** 10.1186/s40104-020-0427-5

**Published:** 2020-03-10

**Authors:** Dong Dai, Shu-geng Wu, Hai-jun Zhang, Guang-hai Qi, Jing Wang

**Affiliations:** Laboratory of Quality & Safety Risk Assessment for Animal Products on Feed Hazards (Beijing) of the Ministry of Agriculture & Rural Affairs, and National Engineering Research Center of Biological Feed, Feed Research Institute, Chinese Academy of Agricultural Sciences, 12 Zhongguancun South St., Haidian District, Beijing, 100081 China

**Keywords:** Development, *In ovo* feeding, Intestinal microbiota, *L*-arginine, Layer chick, Metabolomics

## Abstract

**Background:**

Prenatal nutrition is crucial for embryonic development and neonatal growth, and has the potential to be a main determinant of life-long health. In the present study, we used a layer chick model to investigate the effects of *in ovo* feeding (IOF) of *L*-arginine (Arg) on growth, intestinal development, intestinal microbiota and metabolism. The treatments included the non-injected control, saline-injected control, and saline containing 2, 6, or 10 mg Arg groups.

**Results:**

IOF Arg increased early intestinal index and villus height, and enhanced uptake of residual yolk lipid, contributing to subsequent improvement in the early growth performance of chicks. Prenatal Arg supplementation also increased the early microbial α-diversity, the relative abundance of Lactobacillales and Clostridiales, and decreased the relative abundance of Proteobacteria of cecum in chicks. Furthermore, the shift of cecal microbiota composition and the colonization of potential probiotics were accelerated by IOF of Arg. Simultaneously, metabolomics showed that metabolisms of galactose, taurine-conjugated bile acids and lipids were modulated to direct more energy and nutrients towards rapid growth of intestine at the beginning of post-hatch when embryos received IOF of Arg.

**Conclusions:**

Prenatal Arg supplementation showed beneficial effects on the early intestinal development, cecal microbiota and host metabolism of layer chicks, contributing to subsequent improvement in the early growth performance. These findings provide new insight into the role of IOF of Arg in the establishment of the gut microbiota of newly-hatched layer chicks, and can expand our fundamental knowledge about prenatal nutrition, early bacterial colonization and intestinal development in neonate.

## Introduction

Prenatal nutrition is crucial for embryonic development and neonatal growth, and has the potential to be a main determinant of life-long health [1]. Chicken system has been recently shown to be an excellent model for studying embryonic development of animals [2]. Similar to mammals, the last few days pre-hatch and the first few days post-hatch are recognized as the most critical period for the intestinal development of the chick as well as the early establishment of gut microbiota [3, 4]. Besides, the establishment of an intestinal microbial community is also essential for early intestinal development [5] and recognized for its critical role in the early life programming [6]. In mammals, the recruitment and colonization of microbiota in the digestive tract of a neonate is affected largely by maternal gastrointestinal tract (GIT) microbiota transmission and breast milk composition, such as birth canal and maternal vaginal microbe composition [4]. Unlike the mammal, chick embryo development is out of the strong control of maternal effects during hatching, which provides a good experimental model to study the effects of early nutrient intervention during this neonatal period [6, 7]. With the *in ovo* method, it is also practicable to identify the specific effects of nutrients supply on neonatal growth, organ development and microbiota colonization in a relative separate system [8].

*In ovo* feeding (IOF), the administration of exogenous nutrients into the amnion of the late-term avian embryo, was initially utilized to compensate for the nutrient deficiency caused by metabolic shifts and delay in feeding, and further improve the performance of newly hatched chicks [9]. Among the nutrient supplements, arginine (Arg) is an essential amino acid for gestating mammals and crucial for intestinal development and neonatal growth [1, 10], and markedly increased demands for Arg was observed in pregnancy [11]. Besides, Arg has been applied for the optimal development of the GIT as one of the essential amino acids in poultry [12–14]. Arg is well-known for the physiological and biomedical functions via its metabolites such as nitric oxide (NO) or polyamine [10]. IOF of Arg has also been shown to improve intestinal development and the immunological barrier function, by suppressing the iNOS gene methylation and activating the Arg-NO signaling pathway [15]. In addition, Arg could upregulate the gene expression of mTOR cell signaling pathway which increased enterocytes protein synthesis [12]. These data indicate that multiple mechanisms are responsible for the effects of IOF of Arg on intestinal development. However, few studies have been conducted the effects of IOF of Arg on the intestinal development of layer chicks. The processes of intestinal development were similar in both broiler and layer chicks, although the growth was more rapid in the heavy strain [16]. Due to the long feeding period of layers, the early development of intestine is vital for the later performance [17]. And it is still unclear that the changes in the metabolic profiles induced by IOF of Arg. There is also little information concerning the role of prenatal Arg supplementation in neonatal growth and intestinal development based on the metabolic levels. Besides, *in ovo* administration of bacterial candidates, prebiotics and synbiotics have been shown to stimulate intestinal environment, trigger gut-associated lymphoid tissue maturation and alter the microbial population [18–20]. Another question is whether prenatal Arg supplementation affects the initial microbiome colonization which needs to be explored.

The objective of this study was initially to determine the effects of IOF of Arg on intestinal development, growth performance, cecal microbial populations in layer chicks during the early stage. Metabolomics were then applied to characterize the metabolite changes induced by IOF of Arg. Our findings may contribute to explore the potential benefits of IOF of Arg, and expand our knowledge concerning prenatal Arg nutrition, early bacterial colonization and intestinal development in neonate.

## Materials and methods

### Incubation

All experimental protocols for this study were approved by Animal Care and Use Committee of the Feed Research Institute of the Chinese Academy of Agricultural Sciences in accordance with the Chinese guidelines for animal welfare and experimental protocol. A total of 1,800 fertile eggs with an average weight of 60.81 g were supplied by Beijing Huadu Group Co., Ltd. (Beijing, China) from Jinghong layers of 47 week of age. The replicates (45 eggs on a tray) were distributed homogenously in the automatic-controlled incubator (Chengdu Beili Agricultral Technology Co., Ltd. Chengdu, China) according to standard hatchery procedures (37.8 °C ± 0.1 °C and 60% relative humidity). At 7 and 16 d of incubation, unfertilized and nonviable eggs were taken out following illumination.

### Treatment solutions and IOF procedures

*L*-Arg (Beijing Biotopped Science & Technology Co., Ltd. Beijing, China) and saline (0.85% NaCl) solutions were freshly prepared at 17.5 d of incubation. All injected solutions were sterilized by filtration through a 0.2 μm syringe filter (PTFE, 25 mm, Scientific Strategies, Yukon, OK, USA) and then kept in the incubator for 2 h before injection. To avoid subsequent contamination, the surrounding environment and the surface of the injection site on the larger end of the egg was disinfected with 75% ethanol. Then a small tiny hole was drilled on the injection site. Except for 344 eggs in the non-injected control group (NC), others was *in ovo*-injected with 1 of the 4 solutions: 0.1 mL saline-injected control group (SC), and 0.1 mL saline containing 2, 6, or 10 mg Arg groups (Arg2, Arg6, Arg10) with a 21-gauge needle inserted into the amniotic fluid. The injection process for all eggs was finished within 1.5 h.

### Animal and housing conditions

All healthy and lively female chicks from one treatment were pooled and weighed, while unhatched eggs were recorded to calculate hatchability at the end of the incubation period. After hatching, a total of 120 female layer chicks from each of the 5 experimental treatments with similar body weight (BW) were randomly allotted into 8 replicates (each replicate in one cage). All birds were housed in the thermostatically controlled brooding chicken room which the temperature maintained at 35~36 °C for the first week and was decreased by 2 °C each week until it reached 25 °C. During the 3 days and the next 4 days of the first week, 24 and 22 h energy-saving lighting was provided respectively. From the second week onward, the illumination time was gradually decreased by 2 h each week. The same corn-soybean meal basal diets (Additional file 1: Table S1) and water were supplied ad libitum. The chicks were raised until d 42.

### Histological examination of intestine

On d 3, 14 and 42 after hatching, tissue samples were excised from the middle of duodenum, jejunum and ileum for morphological measurement. Formalin-fixed (10%) samples were prepared using paraffin embedding procedures by sectioning 5 μm and stained with haematoxylin and eosin. A total of 15 intact, well-oriented crypt-villi units per sample were randomly selected and measured. The villus height (VH, from the tip of the villus to the crypt opening) and crypt depth (CD, from the base of the crypt to the level of the crypt opening) were determined using an image processing and analyzing system (Inverted microscope: NIKON CI-S, Tokyo, Japan; Imaging system: NIKON DS-U3, Tokyo, Japan).

### DNA extraction and PCR amplification of 16S rRNA gene sequences

On d 3, 14 and 42 after hatching, microbial DNA was extracted from 0.3 g cecal content samples taken from the NC, Arg6 and Arg10 groups using the E.Z.N.A Soil DNA Kit (Omega Bio-tek, Norcross, GA, USA) according to manufacturer’s instructions. Samples were measured by 1% agarose gel electrophoresis and Nanodrop D-1000 spectrophotometer (Thermo Fisher Scientific, Waltham, MA, USA) to assess integrity and quantity of extracted DNAs. Using the isolated DNA as a template, the V3-V4 hypervariable region of the bacterial 16S rRNA gene was PCR amplified using 5′-ACTCCTACGGGAGGCAGCA-3′ with barcode (forward primer: 338F) and 5′-GGACTACHVGGGTWTCTAAT-3′ with barcode (reverse primer: 806R). The PCR reaction conditions were: initial denaturation at 95 °C for 2 min, followed by 25 cycles consisting of denaturation at 95 °C for 30 s, annealing at 55 °C for 30 s, and extension at 72 °C for 30 s, with a final extension of 5 min at 72 °C. Amplicons were extracted from 2% agarose gels and purified using the AxyPrep DNA Gel Extraction Kit (Axygen Biosciences, Union City, CA, USA) to remove excess primer dimers and dNTPs according to the manufacturer’s instructions. Purified amplicons were pooled in equal amounts and paired-end sequenced (2 × 250 bp) throughput analysis was performed at Shanghai Majorbio Biopharm Biotechnology Co., Ltd., using the Illlumina MiSeq platform. The raw reads were deposited into the NCBI Sequence Read Archive (SRA) database (Accession Number: PRJNA564927).

### Metabolomic profiling

On d 3 and 42 after hatching, 1 bird each replicate with average BW was selected from the NC group and Arg10 group for blood sampling after a 12 h fast. Blood samples were drawn from wing veins, and then centrifuged (3,000×*g *for 10 min) at 4 °C to obtain serum, and stored at − 80 °C before metabolomics analysis.

Metabolites analysis was performed using an ultra performance liquid chromatography (UPLC) system (Waters Corporation, Milford, MA, USA) with a Waters Atlantis T-3 column (100 mm × 2.1 mm; 1.8-μm particle size) at 35 °C and an injection volume of 5 μL. The UPLC system was coupled with a high-resolution tandem mass spectrometer Xevo G2-XS QTOF (MS) (Waters Corporation, Milford, MA, USA). The mobile phases (flow rate of 0.5 mL/min) consisted of 0.1% formic acid (v/v) in double-distilled water (eluent A) and 0.1% formic acid (v/v) in acetonitrile (eluent B). The UPLC system was used to separate chromatogram and the MS was used to detect metabolites eluted form the column in both positive and negative ion modes. For positive ion mode, the capillary and sampling cone voltages were set at 3.0 kV and 40.0 V, respectively. For negative ion mode, the capillary and sampling cone voltages were set at 2.0 kV and 40.0 V, respectively. The mass spectrometry data were acquired in Centroid MSE mode. The TOF mass range was from 50 to 1200 Da and the scan time was 0.2 s. For the MS/MS detection, all precursors were fragmented using 20–40 eV, and the scan time was 0.2 s. During the acquisition, the LE signal was acquired every 3 s to calibrate the mass accuracy. Furthermore, in order to evaluate the stability of the UPLC-MS during the whole acquisition, a quality control sample (Pool of all samples) was acquired after every 10 samples.

### Bioinformatic and statistical analyses

Data analysis of performance, intestinal index, histology-gut integrity and differential species identified were performed using SAS Version 9.2 (SAS Institute Inc., Cary, NC, USA). The replicate (each replicate in one cage) was taken as an experimental unit for analysis of growth performance data, and individual bird was the experimental unit for other parameters. Data were analyzed using one-way ANOVA and means were compared using Duncan’s multiple range test. The linear and quadratic effects of Arg dosage were assessed using regression analysis. Arcsine transformation was used before hatchability data statistical analysis. Differences were considered statistically significant at *P* < 0.05. Data were expressed as mean and pooled SEM.

For microbial community profiling, raw pair-end sequences were demultiplexed and quality-filtered using The Quantitative Insights Into Microbial Ecology (QIIME, version 1.17) [21]. Sequences with length shorter than 150 bp, average Phred scores lower than 20 or containing ambiguous bases were removed. Only sequences that overlap at least 10 bp were assembled using FLASH [22] according to their overlap sequence. Besides, the chimera sequences were identified and removed to obtain effective tags by using the UCHIME [23]. Then the remaining high-quality sequences were clustered into operational taxonomic units (OTUs) with 97% sequence identity by UPARSE (v7.1) [24]. For rarefaction curves and α-diversity analysis (Shannon, Simpson, ACE and Chao1 indices) were calculated using QIIME [21]. β-diversity was estimated by computing the Weighted UniFrac distance and visualized using principal coordinate analysis (PCoA), and the results were plotted using “vegan” and “ggplot2” package in R software (Version 3.4.4). The significance of differentiation of microbial structure among groups was assessed by ANOSIM using R package “vegan” (Additional file 1: Table S2) [25]. Linear discriminant analysis (LDA) effect size (LEfSe) identified were determined to identify the difference in microbial compositions among groups using the non-parametric factorial Kruskal-Wallis test with an alpha value of 0.05 and LDA score of 2.0.

For metabolic profiling, the acquired raw data were processed by Progenesis QI software package (Progenesis QI Version 2.2, Nonlinear Dynamics, Newcastle, UK) for alignment and filtration. Spectral deconvolution and normalization to the total ion amount generated a date matrix involving tR, m/z, and normalized peak area. The analytical variation was corrected with the quality control-based (QC) robust LOESS signal correction algorithm. A threshold of 30% was set for the relative standard deviation values of metabolites in the QC samples. All data were generalized logarithm-transformed and Pareto scaled before multivariate statistical analysis, which included an unsupervised principal-component analysis (PCA) and partial least squares discriminant analysis (PLS-DA). The different metabolites were determined by the combination of the VIP value > 1 of PLS-DA model and the *P* values (< 0.05) from Kruskal-Wallis test on the normalized peak intensities. Fold change was calculated as binary logarithm of average normalized peak intensity ratio between two groups. HMDB and KEGG databases were used to check and confirm the differential metabolites.

## Results

### Hatchability and body weight

As shown in Table 1, significant differences in hatchability and hatching weight among groups were not observed in response to IOF of Arg (*P* > 0.05), suggesting all chicks remained in good health during the pre-challenging period. There were no significant differences in BW of 7 and 14-day-old chicks. But IOF of Arg increased the BW of 21-day-old chicks in the Arg10 group compared to the NC group (*P* = 0.039). On d 42, compared with the NC group, the BW were significantly increased in Arg6 and Arg10 groups (*P* = 0.042).

**Table 1:** Effects of *in ovo* feeding of *L*-arginine on hatchability, body weight of layer chicks

Item			Arg dosage (mg/egg)			Pooled SEM	*P*-value		
	NC^1^	SC^2^	2	6	10		ANOVA	Linear	Quadratic
Hatchability, %	91.36	92.72	93.67	91.91	91.00	0.67	0.518	0.910	0.777
HW^3^, g	42.50	42.34	43.26	42.30	42.19	0.15	0.137	0.297	0.379
BW^4^, g									
d 7	73.38	74.67	75.94	74.37	74.97	0.35	0.220	0.665	0.827
d 14	122.42	125.89	126.02	124.35	126.95	0.82	0.474	0.529	0.795
d 21	174.60^b^	177.81^ab^	178.56^ab^	181.00^ab^	183.85^a^	1.03	0.039	0.018	0.382
d 42	434.29^b^	444.65^ab^	444.72^ab^	452.60^a^	452.28^a^	2.21	0.042	0.202	0.301

^a–b^Means within a row with no common superscripts differ significantly (*P* < 0.05). ^1^NC, non-injected control group; ^2^SC, saline (0.85% NaCl)-injected control group. ^3^HW, hatching weight; ^4^BW, body weight. Data are the mean of 8 replicates. Orthogonal polynomial contrasts were used to determine the linear and quadratic effects of increasing concentrations of *L*-arginine solution

### Growth performance and development of digestive organs

The effects of IOF of Arg on growth performance of layer chicks were showed in Table 2. No linear and quadratic effects were observed in growth performance during d 1 to 14 (*P* > 0.05). However, we found that the ADG (*P* = 0.021, *P* = 0.032, respectively) and ADFI (*P* = 0.037, *P* = 0.044, respectively) during d 15 to 21 were linearly and quadratically increased by IOF of Arg. Higher ADG (*P* = 0.030) and ADFI (*P* = 0.021) during d 1 to 21 were also observed in the Arg10 group. Simultaneously, we also found that IOF of Arg linearly and quadratically decreased F/G (*P* = 0.047, *P* = 0.032, respectively).

**Table 2:** Effects of *in ovo* feeding of *L*-arginine on growth performance of layer chicks

Item^3^			Arg dosage (mg/egg)			Pooled SEM	*P*-value		
	NC^1^	SC^2^	2	6	10		ANOVA	Linear	Quadratic
d 0 to 14									
ADG, g	6.14	6.40	6.35	6.29	6.56	0.07	0.451	0.757	0.568
ADFI, g	11.45	12.11	11.55	11.60	11.85	0.12	0.410	0.109	0.279
F/G	1.87	1.90	1.82	1.82	1.87	0.02	0.500	0.442	0.714
d 15 to 21									
ADG, g	7.86^b^	7.62^b^	7.62^b^	7.70^b^	8.56^a^	0.10	0.011	0.021	0.032
ADFI, g	19.42^b^	19.62^b^	19.19^b^	19.72^b^	20.82^a^	0.16	0.019	0.037	0.044
F/G	2.53	2.58	2.49	2.50	2.41	0.02	0.213	0.622	0.535
d 0 to 21									
ADG, g	6.45^b^	6.71^ab^	6.69^ab^	6.72^ab^	7.00^a^	0.06	0.030	0.073	0.143
ADFI, g	13.80^b^	14.61^a^	14.25^ab^	14.02^ab^	14.66^a^	0.11	0.021	0.847	0.075
F/G	2.14^a^	2.14^a^	2.13^a^	2.08^b^	2.07^b^	0.01	0.016	0.047	0.032
d 22 to 42									
ADG, g	12.23	12.65	12.55	12.86	12.52	0.09	0.228	0.869	0.647
ADFI, g	32.30	32.37	32.80	33.49	33.00	0.22	0.075	0.545	0.284
F/G	2.62	2.61	2.62	2.61	2.64	0.01	0.957	0.581	0.833
d 0 to 42									
ADG, g	9.28	9.55	9.51	9.55	9.58	0.05	0.400	0.509	0.826
ADFI, g	22.49	22.67	22.25	23.16	23.05	0.15	0.291	0.395	0.427
F/G	2.45	2.42	2.40	2.41	2.41	0.01	0.564	0.717	0.935

^a–b^Means within a row with no common superscripts differ significantly (*P* < 0.05). Data are the mean of 8 replicates. ^1^NC, non-injected control group; ^2^SC, saline-injected control group. ^3^ADG, average daily gain; ADFI, average daily feed intake; F/G, feed conversion ratio (feed: gain, g: g). Orthogonal polynomial contrasts were used to determine the linear and quadratic effects of increasing concentrations of *L*-arginine solution

This experiment proved that no significant difference in index of proventriculus and gizzard in response to IOF of Arg, but a markedly reduced yolk sac index on d 0 (*P* = 0.008) (Table 3). IOF of Arg linearly and quadratically increased duodenum index (*P* = 0.010, *P* = 0.035, respectively) of 3 d-old and jejunum index (*P* = 0.025, *P* = 0.039, respectively) of 14 d-old chicks. On d 42, IOF of Arg linearly and quadratically increased jejunum index (*P* = 0.031, *P* = 0.042, respectively).

**Table 3:** Effects of *in ovo* feeding of *L*-arginine on digestive organ index of layer chicks^1^

Item^2^			Arg dosage (mg/egg)			Pooled SEM	*P*-value		
	NC	SC	2	6	10		ANOVA	Linear^3^	Quadratic^3^
d 0 Index, %									
Yolk sac	14.52^a^	14.49^a^	11.05^b^	11.72^b^	11.94^b^	0.40	0.008	0.551	0.282
Proventriculus	0.53	0.56	0.66	0.54	0.60	0.02	0.086	0.656	0.910
Gizzard	4.01	4.35	4.48	4.23	4.53	0.08	0.211	0.352	0.800
Duodenum	16.34	15.70	15.56	15.77	16.15	0.20	0.721	0.294	0.602
Jejunum	28.16^b^	28.68^b^	34.49^a^	29.61^b^	31.79^ab^	0.73	0.020	0.928	0.841
Ileum	25.40	25.54	26.34	25.40	25.99	0.64	0.989	0.910	0.896
d 3 Index, %									
Yolk sac	2.67	2.91	1.93	1.89	1.85	0.16	0.088	0.065	0.159
Proventriculus	1.16	1.17	1.18	1.23	1.21	0.02	0.625	0.857	0.204
Gizzard	6.52	6.25	6.54	6.62	6.57	0.07	0.536	0.611	0.207
Duodenum	22.48^b^	22.67^b^	22.93^ab^	24.71^ab^	25.39^a^	0.40	0.049	0.010	0.035
Jejunum	42.13	41.07	41.73	44.70	42.61	0.57	0.323	0.467	0.112
Ileum	31.23	32.98	34.75	36.36	33.92	0.74	0.224	0.803	0.157
d 14 Index, %									
Proventriculus	0.77	0.71	0.76	0.80	0.74	0.01	0.100	0.546	0.131
Gizzard	3.63	3.57	3.68	3.62	3.66	0.05	0.971	0.674	0.924
Duodenum	10.70	10.65	9.39	9.22	10.71	0.40	0.606	0.822	0.272
Jejunum	19.02^b^	19.21^b^	22.52^a^	22.05^a^	23.66^a^	0.45	0.001	0.025	0.039
Ileum	18.56	17.58	20.07	19.94	20.93	0.42	0.089	0.040	0.075
d 21 Index, %									
Proventriculus	0.71	0.67	0.69	0.71	0.68	0.01	0.466	0.525	0.284
Gizzard	2.99	2.84	2.74	2.91	2.95	0.04	0.365	0.741	0.879
Duodenum	7.82	7.73	7.38	7.67	7.51	0.08	0.503	0.702	0.975
Jejunum	15.37	16.36	15.10	16.21	14.24	0.27	0.108	0.547	0.651
Ileum	14.51	15.92	14.54	15.17	13.24	0.30	0.109	0.777	0.305
d 42 Index, %									
Proventriculus	0.69	0.71	0.63	0.72	0.71	0.02	0.308	0.422	0.578
Gizzard	2.60	2.47	2.44	2.57	2.43	0.05	0.740	0.866	0.701
Duodenum	4.13	3.85	3.88	4.10	3.97	0.05	0.194	0.328	0.229
Jejunum	8.43^b^	7.89^b^	7.77^b^	8.04^b^	9.21^a^	0.13	0.001	0.031	0.042
Ileum	7.72^a^	7.17^ab^	6.58^b^	7.54^a^	7.80^a^	0.12	0.004	0.235	0.103

^a–b^Means within a row with no common superscripts differ significantly (*P* < 0.05). ^1^ Data are the mean of 8 replicates. NC, non-injected control group; SC, saline-injected control group.^2^ Yolk sac and stomach index, % = absolute weight, g / body weight, g × 100, Intestinal index, % = absolute length, cm/ body weight, g × 100. ^3^Orthogonal polynomial contrasts were used to determine the linear and quadratic effects of increasing concentrations of *L*-arginine solution

### Intestinal histomorphology

**Fig. 1:**
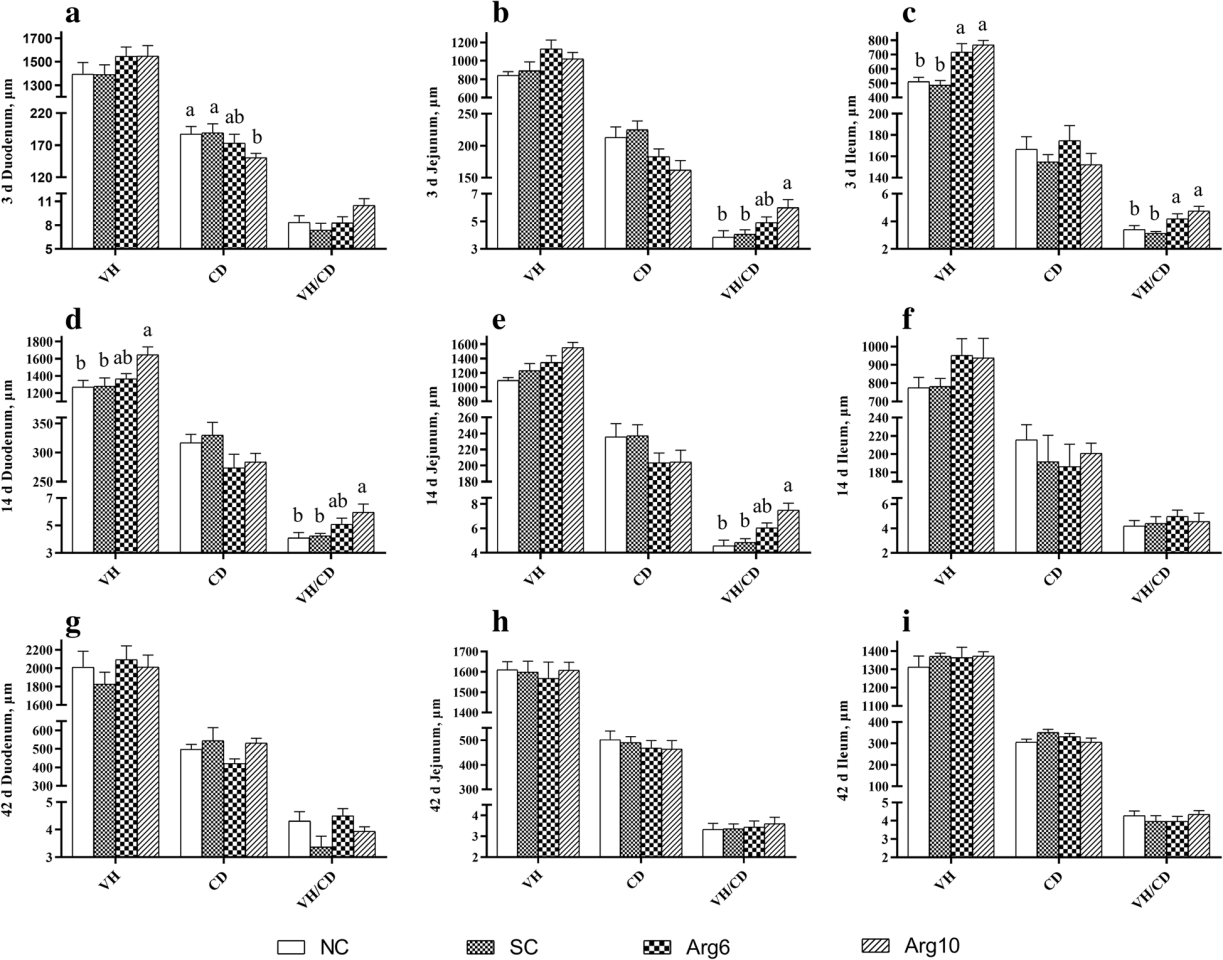
Effects of *in ovo* feeding of *L*-arginine on intestinal morphological structure of layer chicks. **A**, **D** and **G** were the duodenum on d 3, 14 and 42 respectively; **B**, **E** and **H** were the jejunum on d 3, 14 and 42 respectively; **C**, **F** and **I** were the ileum on d 3, 14 and 42 respectively. ^a-b^Values at the same index with no common superscripts differ significantly (*n* = 8; *P* < 0.05). NC, non-injected control group; SC, saline-injected control group; Arg6, injected with 6 mg Arg; Arg10, injected with 10 mg Arg

The intestinal histomorphology is considered as the most obvious symbol of intestinal development (Additional file 1: Figure S1). Duodenum CD of the Arg10 group was reduced than that in NC and SC groups on d 3 (*P* = 0.046, Fig. 1a), and duodenum VH was increased than that in NC and SC groups on d 14 (*P* = 0.041, Fig. 1d). The greater villus height: crypt depth ratio (VH/CD) of jejunum was observed in the Arg10 group than that in NC and SC groups on d 3 and 14 (*P* = 0.004, *P* = 0.039, Fig. 1b, e, respectively). What’s more, we also found that the ileum VH and VH/CD of Arg groups were higher than that in NC and SC groups on d 3(*P* = 0.003, *P* = 0.001, Fig. 1c, respectively). However, there was no significant effect on the intestinal morphology of chicks on d 42 (*P* > 0.05, Fig. 1g, h, i). In one word, there is a trend that the difference of intestinal morphology narrowed gradually among the groups with age.

### Intestinal microbial diversity and community

**Fig. 2:**
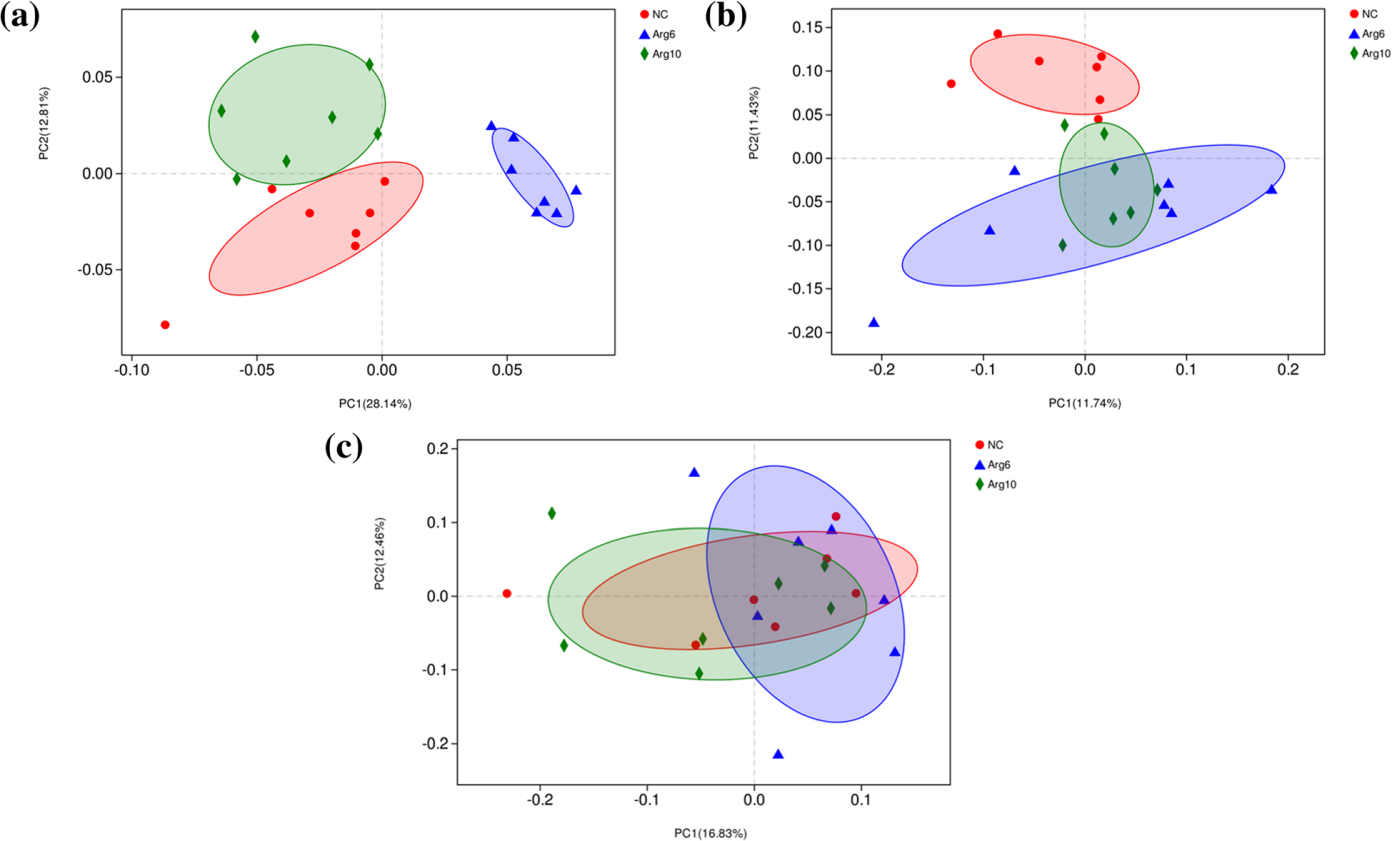
Principal coordinate analysis (PCoA) representing the similarity of cecal microbiota in layer chicks. **a**, **b** and **c** were PCoA results based on weighted unifrac distance on d 3, 14 and 42 respectively. NC, non-injected control group; Arg6, injected with 6 mg Arg; Arg10, injected with 10 mg Arg

After filtering, an average of 53,420 reads per sample was obtained (38,148–74,120). First, sequencing depths were examined by plotting the rarefaction curve for richness and the numbers of shared OTUs. Most of the samples reached plateaus, indicating that sampling depth was adequate. However, one obvious outlier in each group interfering with the microbiota statistical analysis was excluded in the visual multivariate data. Therefore, a total of 7 replicates per group were included in the statistical analysis. In the α-diversity analysis, the Shannon and Simpson indices were used to assess the microbiota diversity, and the ACE and Chao1 estimators reflected the microbiota richness [26]. The results are listed in Table 4. A markedly increased Shannon index (*P* <  0.001) and reduced Simpson index (*P* = 0.004) were observed in Arg6 and Arg10 groups on d 3. The ACE and Chao1 estimators were higher in the Arg6 group than in the NC group (*P* = 0.041, *P* = 0.043). However, no significant difference among groups was observed on d 14 and d 42 (*P* > 0.05). β-diversity analysis were performed to compare the overall microbial profiles of all the groups as displayed in Fig. 2. PCoA analysis was first performed to present a holistic perception of the microbiota. Results for PCoA visually showed that the groups were mainly scattered into three clusters on d 3 (Fig. 2a), which illustrated the microbiota compositions were quite dissimilar to each group. PCoA analysis showed a trend of aggregation of microbial communities on d 14 (Fig. 2b) and the samples from each group were fully aggregated on d 42 (Fig. 2c).

**Table 4:** Effects of *in ovo* feeding of *L*-arginine on cecal microbial diversity of layer chicks

Item^1^	NC	Arg6	Arg10	Pooled SEM	*P*-value
3 d					
Shannon	1.77^b^	2.38^a^	2.34^a^	0.08	< 0.001
Simpson	0.28^a^	0.18^b^	0.16^b^	0.02	0.004
ACE	98.23^b^	126.66^a^	111.20^ab^	4.91	0.041
Chao1	94.31^b^	115.66^a^	99.39^ab^	3.81	0.043
14 d					
Shannon	3.37	3.31	3.63	0.09	0.314
Simpson	0.10	0.11	0.07	0.01	0.291
ACE	256.11	265.12	254.81	5.21	0.711
Chao1	263.88	265.07	258.82	5.45	0.893
42 d					
Shannon	3.48	3.76	3.37	0.10	0.307
Simpson	0.12	0.09	0.11	0.01	0.571
ACE	450.28	457.42	460.07	5.92	0.779
Chao1	453.91	455.76	460.65	7.22	0.925

^a–b^Means within a row with no common superscripts differ significantly (*P* < 0.05). Data are the mean of 7 replicates. ^1^NC, non-injected control group; Arg6, injected with 6 mg *L*-arginine; Arg10, injected with 10 mg *L*-arginine

**Fig. 3:**
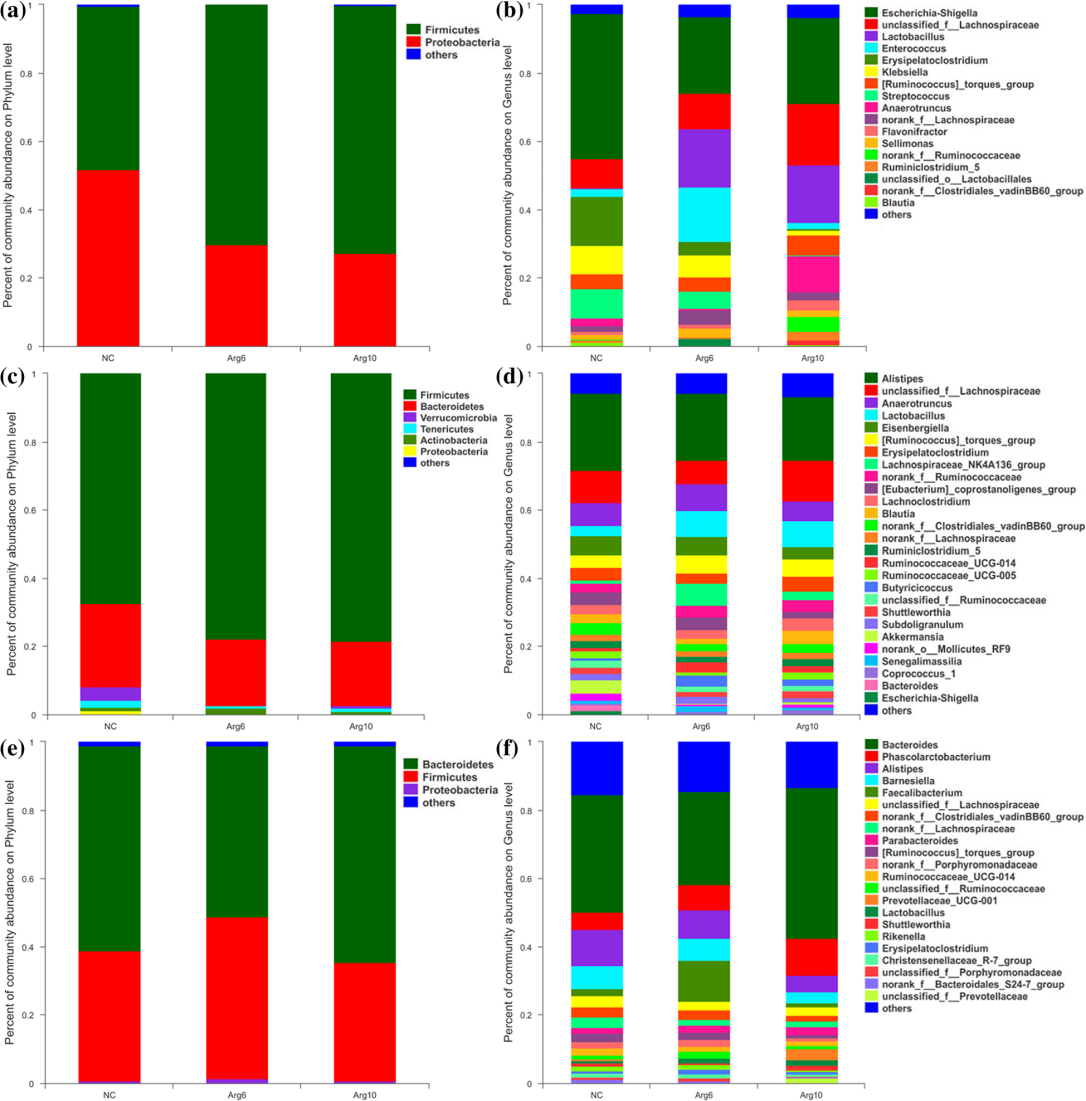
Composition of cecal microbiota of layer chicks at phylum and genus level. **a** and **b**, on d 3. **c** and **d**, on d 14. **e** and **f**, on d 42. NC, non-injected control group; Arg6, injected with 6 mg Arg; Arg10, injected with 10 mg Arg

**Fig. 4:**
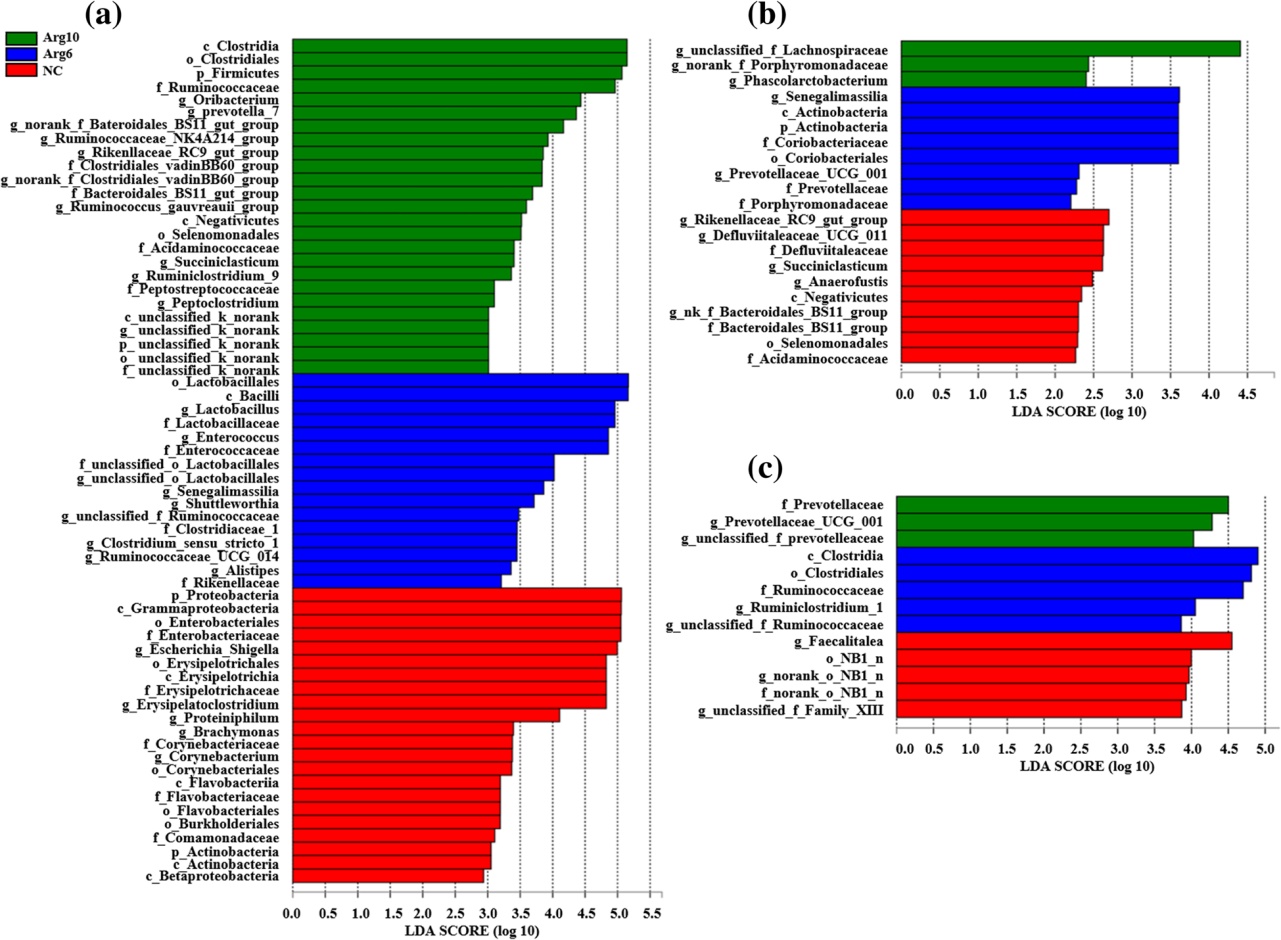
Linear discriminant analysis (LDA) effect size (LEfSe) of cecal microbiota in layer chicks. **a**, **b** and **c** were LEfSe results on d 3, 14 and 42 respectively. NC, non-injected control group; Arg6, injected with 6 mg Arg; Arg10, injected with 10 mg Arg

To assess the differences induced by IOF of Arg in the bacterial community members of the cecal microbiota, taxonomic compositions were analyzed at the phylum and genus levels. The changes of early cecal microbiota for layer chicks were revealed, which two (Firmicutes and Proteobacteria), six (Firmicutes, Bacteroidetes, Verrucomicrobia, Tenericutes, Actinobacteria and Proteobacteria) and two (Bacteroidetes and Firmicutes) major phylum-level phyla dominated bacterial community on d 3 (Fig. 3a), 14 (Fig. 3c) and 42 (Fig. 3e) respectively. Compared with the NC group, Arg6 and Arg10 groups were characterized by higher relative abundance of Firmicutes (70.20 and 72.67%, respectively; *P* = 0.013), lower levels of Proteobacteria (29.71 and 27.21%, respectively; *P* = 0.020) on d 3. Compared with bacterial compositions of d 3 and 14 at genus level, we found that the colonization time of cecal microbiota for *Lactobacillus*, *norank_f_Ruminococcaceae* and *Clostridiales_vadinBB60_group* in Arg groups were earlier than that in the NC group (Fig. 3b, d). The changes in the composition of the cecal microbiota were also explored by the LEfSe method. The results represented the cecal microbiota with the predominant bacteria in NC, Arg6 and Arg10 groups, which more bacterial taxa were found to discriminate different groups from the samples on d 3 (Fig. 4a) than that of d 14 (Fig. 4b) and 42 (Fig. 4c). On d 3 post-hatch, the phyla of Firmicutes was enriched in the Arg groups while Proteobacteria was found to be enriched in the NC group. At the genus level, *Lactobacillus* and *Enterococcus* belonging to the order Lactobacillales, *Shuttleworthia*, *Ruminococcaceae_UCG_014* and *Clostridium_sensu_stricto_1* belonging to the order Clostridiales, were observed to be enriched in the Arg6 group. In the Arg10 group, bacteria identified as *Ruminiclostridium_9*, *Peptoclostridium*, *norank_f_Clostridiales_vadinBB60_group* and *Oribacterium*, which affiliated to the order Clostridiales became enriched in the ceca similarly. Besides, other bacterial taxa with relative low abundance also were enriched in the Arg10 group, including *Prevotella_7*, *norank_f_Bacteroidales_BS11_gut_group* and *Rikenellaceae_RC9_gut_group*. On d 14, LEfSe indicated that the increased relative abundance of *unclassified_f_Lachnospiraceae* and *Senegalimassilia* was associated with IOF of Arg groups. Besides, *Prevotellaceae_UCG_001* and *Phascolarctobacterium* were also higher in Arg6 and Arg10 groups respectively (Fig. 4b). However, on d 42, we only found that Ruminococcaceae family (*Ruminiclostridium_1* and *unclassified_f_Ruminococcaceae*), Prevotellaceae family (*Prevotellaceae_UCG_001* and *unclassified_f_Prevotellaceae*) were linked to the increased relative abundance in the groups with IOF of Arg (Fig. 4c). In addition, differential species identified from cecal microbiota from different groups by using the one-way ANOVA were showed in Additional file 1: Figure S2.

### Correlations between microbiota and intestinal morphology

**Fig. 5:**
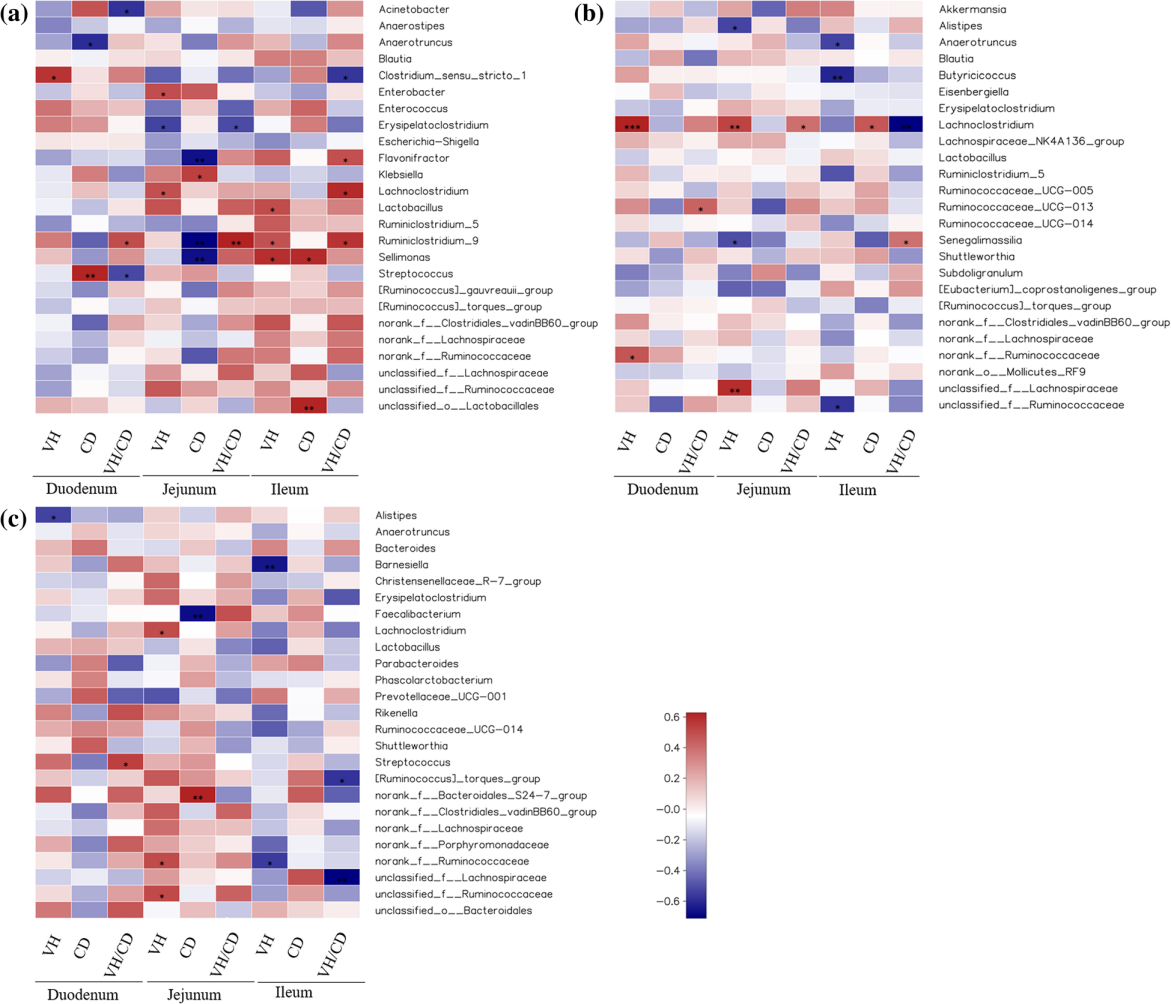
Heatmap of spearman’s correlation between intestinal microbiota and intestinal morphology. **a**, **b** and **c** were correlation results base on the relative abundances of 25 key phylotypes at genus level on d 3, 14 and 42 respectively. The colors range from blue (negative correlations) to red (positive correlations). Significant correlations are noted by 0.01 < *P* ≤ 0.05 *, 0.001 < *P* ≤ 0.01**, *P* ≤ 0.001***

To explore the particular bacteria associated with intestinal development, correlations between abundance of microbiota and intestinal morphology was analyzed based on the Spearman’s correlation coefficients (Fig. 5). The heatmap reflected significant positive correlations between intestinal development (higher VH, shorter CD and lager VH/CD indicate superior development) and *Anaerotruncus*, *Clostridium_sensu_stricto_1*, *Enterobacter*, *Flavonifractor*, *Lachnoclostridium*, *Lactobacillus* and *Ruminiclostridium_9* on d 3 (*P* <  0.05). In contrast, significant negative correlations between intestinal development and *Acinetobacter*, *Erysipelatoclostridium*, *Klebsiella* and *Streptococcus* were also determined from the heatmap (*P* < 0.05) (Fig. 5a). In addition, the abundance of genus *Butyricicoccus*, *Ruminococcaceae_UCG_013* and *Feacalibacterium* were also showed highly positive correlations with the intestinal development, while *Alistipes*, *Senegalimassilia* and *Barnesiella* were negatively correlated on d 14 (Fig. 5b) and 42(Fig. 5c) (*P* < 0.05).

### Metabolomic analysis

To characterize the metabolite changes induced by IOF of Arg, we performed LC-MS/MS-based metabolomic analysis in serum from the birds of NC and Arg10 groups on d 3 and 42. Using principal component analysis (PCA) and partial least squares discriminant analysis (PLS-DA), we observed a clear differentiation of NC and Arg10 groups in ESI-model with two components on d 3 (Additional file 1: Figure S3). There was a same tendency as intestinal morphology and microbiota as presented on the PCA score plots. Approximately 81 differential metabolites were selected based on the criteria of VIP ≥ 1 in PLS-DA analysis, fold change ≥1.2 in fold change analysis and *P* < 0.05 in Kruskal-Wallis test between NC and Arg10 groups. However, only 24 differential metabolites were found after primary identification. A total of 4 metabolites involved in carbohydrate metabolism, 6 and 4 metabolites were related to lipid metabolism and primary bile acid biosynthesis respectively. In addition, we also found that 10 secondary metabolite levels were significantly reduced in the Arg10 group (Table 5).

**Table 5:** Differential metabolites that mapped to pathway on day 3 post-hatch

Differential metabolites	Retention time	m/z	Fold change^a^	*P*-value^b^	VIP^c^	Pathway
Stachyose	0.6476833	701.18808	0.420	0.007	3.454	Galactose metabolism
Raffinose	0.6476833	539.13622	0.555	0.004	2.501	
Manninotriose	0.6476833	539.13622	0.555	0.004	2.501	
Sucrose	0.6334000	377.08445	0.605	0.002	2.146	
Taurine	0.5905500	124.00700	1.577	0.005	1.810	Primary bile acid biosynthesis
Taurochenodeoxycholate	7.2767833	498.28747	0.283	0.004	4.431	
Taurodeoxycholate	8.2390167	498.28884	0.400	0.007	3.579	
Tauroursodeoxycholic acid	6.9417667	498.28752	0.444	0.030	2.550	
Linoleic acid	8.6883500	279.23228	0.796	0.042	1.408	Linoleic acid metabolism
Dihomo-γ-linolenic acid	9.5298333	305.24812	1.414	0.047	1.141	Linoleic acid metabolism
9,10-DHOA	8.4390333	313.23741	2.468	0.027	3.496	Linoleic acid metabolism
12,13-DHOA	7.5975333	313.23730	0.606	0.039	1.442	Linoleic acid metabolism
13,16,19-Docosatrienoic acid	9.8934500	333.27922	4.063	0.038	3.999	Lipid metabolism
DHA ethyl ester	9.5298333	355.26264	1.771	0.034	1.592	
Amaranthin	0.6334000	761.14313	0.384	0.005	3.363	Secondary metabolites
Cucurbitacin D	9.4012667	515.30267	0.339	0.011	4.030	

^a^Fold Change in the Arg10 group compared with the NC group. ^b^*P*-values were calculated from Kruskal-Wallis test. ^c^VIP (variable importance in the projection) values were obtained from partial least squares discriminant analysis models

## Discussion

The potential benefits of IOF of Arg in broilers have been reported recently, such as improving growth performance and development of digestive organs after hatch [27], modulating the release of gastrointestinal hormones [28] and increasing the activities of intestinal digestive enzymes [29]. In the current study, the positive effects of IOF of Arg on growth of layer chicks were not observed until the third week of age after hatch, indicated by the increased BW on d 21 and d 42 (Table 1), while the beneficial effects on intestinal development were only observed in the first 2 weeks (Fig. 1). During the first few days after hatching, growth occurs exclusively in the digestive system of chicks [3]. For example, remaining yolk nutrients were utilized preferentially for intestinal development of chicks rather than body weight gain [7]. This is consistent with the observation that IOF of Arg led to a lower weight of yolk sac in neonatal chicks (Table 3), which indicated an acceleration in utilization of remaining yolk nutrients. Improved early intestinal development, as evidenced by increased intestinal index and modulated intestinal morphology (including VH and VH/CD ratio), could enhance intestinal nutrient digestibility and absorption capacity [30], and may further contribute to subsequent improvement in the early growth performance. However, no significant differences were found on d 22–42, which might be related to more mature and stable intestine microbiota of chicks and the compensatory process.

Given the crucial importance of microbial colonization in neonatal GIT and immune development, nutritional modulation of maternal intestinal microbiota in human for the health of the offspring has attracted considerable attention [31]. However, little information is available regarding the effects of *in ovo* administration on the intestinal microbiota in later stages of bird’s life span [7]. In this study, the distances of PCoA and number of characteristic bacteria among the groups gradually reduced from days 3 to 42 (Fig. 2), implied the effects of IOF of Arg on community composition decreased with the development of gut microbiota. Although the age of chicks was reported to be more influential in intestinal microbiome development especially in the early establishment period, dietary and other environmental factors became more prominent with age [32]. That may be mainly responsible for the minor difference among groups in cecal microbial composition with age. In fact, the establishment of microbial communities in GIT of chicks undergoes several successional stages over the whole production cycle [33]. The early colonization during the first days may be therefore the key period for the *in ovo* administration.

As the control chicks aged from 3 to 42 days old, their cecal microbiota successively dominated by bacteria within the phyla Proteobacteria (51.77%, 3 d), Firmicutes (67.38%, 14 d) and Bacteroidetes (58.86%, 42 d) (Fig. 3a, c, e), similar to previous observation in chicks [34]. It is of interest to notify the marked change of composition of the cecal microbiome caused by *in ovo* administration on d 3. The chicks from IOF of Arg groups had lower abundance of phyla Proteobacteria and higher proportion of Firmicutes as compared to the control. Day-old chicks has been reported to begin with a gut colonized by few bacterial species but characterized by a high prevalence of Enterobacteriaceae, belonging to phyla Proteobacteria [32, 34]. In fact, Proteobacteria has been detected in chick embryos with the abundance of 86%, and may derive from the maternal hens by the process of fertilization and egg formation in the oviduct [35]. But a sharp decline of Enterobacteriaceae occurred during the first week of age after hatch, with the relative abundance of ~ 80% decreased to ~ 30% [32, 34]. The strongly fluctuated community composition might be associated with the infection and gastrointestinal disorders primarily observed to occur within the first week post-hatch [19]. In the current study, higher abundance of Firmicutes at expense of Proteobacteria indicated that IOF of Arg accelerated the shift in cecal microbiome composition from the early dominance of Enterobacteriaceae to Firmicutes-dominated. At the genus level, less *Escherichia-Shigella* belonging to Proteobacteria were observed in Arg groups than the control group (*P* = 0.045; Additional file 1: Figure S2). And increasing abundance of *Lactobacillus*, *Enterococcus*, *Oribacterium*, *Ruminococcaceae_NK4A214_group*, *norank_f_Clostridiales_vadinBB60_group* and *Ruminococcaceae_UCG_014* induced by Arg injection were the main driver of the rise in Firmicutes abundance (~ 23.72%) on d 3, which was similar to the abundance of Firmicutes (~ 10.69%) in control chicks on d 14. According to the bacterial compositions of d 3 and 14 at genus level, we found that *Lactobacillus*, *norank_f_Ruminococcaceae* and *Clostridiales_vadinBB60_group* emerged earlier in Arg groups than the control group. Unexpectedly, the previous study indicated that the growth of *Lactobacillus* was dependent on the Arg to a great extent [33], and the lactic acid produced by *Lactobacillus* could be further used as substrate by butyrate-producing bacteria such as Ruminococcaceae [36]. In addition, Arg can modulate the metabolism of the Arg, serine and aspartate-family of amino acids in intestinal bacteria and decrease the utilization of most amino acids in mixed bacteria [37]. These may partly explain why Arg can stimulated probiotics and affected the microbiota. The above results suggest that the shift of cecal microbiota composition and the colonization of potential probiotics were accelerated by IOF of Arg.

Further analysis revealed more differential bacteria at various taxonomic levels among groups. On d 3 post-hatch, bacteria which belonging to orders Lactobacillales (mainly represented by *Lactobacillus*) and Clostridiales (mainly represented by Ruminococcaceae) were identified as biomarkers to distinguish cecal microbiota of chicks in Arg groups from the control on d 3 post-hatch (Fig. 4a). *Lactobacillus* is one of transiently dominated genera in cecal microbiota of birds, and contributed to the gut defense function by competitive exclusion of intestinal pathogens or via modulation of local cell-mediated immunity [38]. That also may partly explain the reduced abundance of *Escherichia-Shigella* and *Erysipelotruchaceae* caused by IOF of Arg in the current study. Besides, dietary supplementation of *Lactobacillus* improved intestinal morphology and mucosal barrier function in the weaned piglets [39], similar to our observation that the *Lactobacillus* enrichment correlated with mucosal villus height of ileum (Fig. 5a). And increasing levels of *Lactobacillus* in the GIT has been considered as a target to prevent and/or alleviate microbiota-associated diseases [18, 39]. As to Clostridiales, it was observed in the guts of shorebird chicks with high abundance, and inferred a role for commensalism or potentially mutualism in the GIT [40]. The large increment of Clostridiales in the Arg groups was caused by the remarkable increase in Ruminococcaceae. In addition, *Ruminiclostridium_9* affiliated to Ruminococcaceae had highly positive relationships with the VH and VH/CD of intestine on d 3 (Fig. 5a). Studies have shown that *Ruminiclostridium* was associated with the production of short-chain fatty acids (SCFAs) [41], and then can supply energy to the intestinal epithelium directly. Moreover, the underlying mechanism considering that the lactic acid produced by *Lactobacillus* is then further consumed by butyrate-producing bacteria such as Ruminococcaceae to produce butyrate [36]. Especially, Ruminococcaceae is capable of transforming primary bile acids into secondary bile acids via a multi-step 7α-dehydroxylation reaction [42]. This study showed that Arg supplementation triggered increase in Ruminococcaceae concurrent with a reduction of taurochenodeoxycholate, taurodeoxycholate and tauroursodeoxycholic acid, suggesting the intestinal microbiota of birds supplemented Arg became more efficient in regulating primary bile acid metabolism.

Several species were identified as biomarkers to distinguish cecal microbiota of layer chicks among groups on d 14, including *unclassified_f_Lachnospiraceae* and *Senegalimassilia*, which affiliated to the Lachnospiraceae and Coriobacteriaceae family respectively (Fig. 4b). Lachnospiraceae is capable of readily degrading less recalcitrant non-starch polysaccharides and starch to produce butyrate used for intestinal development [43]. In this study, we also found that Lachnospiraceae showed highly positive correlations with VH of the duodenum and jejunum on d 14(Fig. 5b). It has been demonstrated that SCFAs could promote intestinal cell proliferation and modulate intestinal morphological changes [44]. Furthermore, a positive correlation has also been found between the abundance of cecal Lachnospiraceae and feed conversion efficiency in commercial broiler chickens [45]. The recent study has shown that SCFAs could increase the relative mRNA expression of intestinal development-related genes, including insulin-like growth factor-1, insulin-like growth factor-1 receptor, glucagon-like peptide 2, and glucagon-like peptide 2 receptor, shortening the G0G1 phase of intestinal cells, and reduce the abundance of the pro-apoptosis genes [46]. This result might explain highly positive correlations between the SCFAs-producing bacteria and intestinal histomorphology. As to *Senegalimassilia*, the genome of it encoded proteins for glycolysis, e.g. phosphofructokinase as well as sugar ABC transporter indicating some potential metabolic function in sugar utilization [47]. *Prevotellaceae_UCG_001*, *Ruminiclostridium_1*, *Faecalitalea* were identified as biomarkers to distinguish cecal microbiota of chicks among groups on d 42 (Fig. 4c). *Prevotella* possesses enzymes that can degrade cellulose and xylan to produce propionate used for host [48]. What’s more, the previous study reported that the abundance of *Prevotellaceae_UCG_001* was negatively correlated with the blood parameters about glucose and lipid [49]. It was thus speculated that *Prevotellaceae_UCG_001* might contribute to the host glucose and lipid metabolism through the production of propionate. In addition, NC group was enriched by *Faecalitalea* that few studies have reported before in chickens. However, *Faecalitalea* affiliates to Erysipelotrichaceae family, which was known for relation with inflammation disorders [50]. Together these data reveal potential effects of IOF of Arg on intestinal microbial community, and suggest that microbial biomarkers were associated with intestinal development.

Metabolic pathways potentially affected by IOF of Arg were identified by the use of metabonomic analysis of serum metabolites profiles, which had not been investigated before. It was interesting to note that the decreased effects of IOF of Arg on metabolic changes with age was similar to the observation on microbial composition. A prominent metabolic change caused by IOF was only observed on d 3 post-hatch, while there was no obvious group separation on d 42 post-hatch. Firstly, IOF of Arg affected the levels of several metabolites related to lipid metabolism on d 3 post-hatch. Neonatal chicks preferentially absorbed the phosphoglycerides fraction that is relatively rich in essential omega-6 and omega-3 fatty acids from residual yolk sac for intestinal development [51–53]. Increased serum levels of docosahexaenoic acid ethyl ester and 13,16,19-docosatrienoic acid (Table 5) in the current study may result from the uptake of residual yolk lipid. In addition, an extensive lipogenic capacity was rapidly established in liver of post-hatch chicks in order to accommodate metabolic changes. For example, linoleic acid desaturation in the liver increased with the approach of hatching [54], and hepatic proportions of linoleic acid in chicks decreased substantially during 3 days after hatch [55], which suggested an importance of linoleic acid metabolism in newly hatched chicks. In this study, the serum levels of linoleic acid decreased in the Arg injection group, while the levels of dihomo-γ-linolenic acid increased. Dihomo-γ-linolenic acid is an intermediate metabolite in arachidonic acid synthesis from linoleic acid. Collectively, the changes of metabolites involved in lipid metabolism may indicate an enhanced uptake of residual yolk lipid and modulated linoleic acid metabolism in liver, which could contribute to the potential benefits of IOF of Arg on early intestinal development and growth. Another series of putative annotated metabolites involved in galactose metabolism were differentially abundant. In fact, Arg plays an important role in regulating energy metabolism [56], which can stimulate the release of hormones, such as glucagon, to accelerate the conversion of galactose to glucose [57, 58], and then may reduce the synthesis of oligosaccharides. In addition, a negative correlation between the levels of serum oligosaccharides and the abundance of Ruminococcaceae (R = − 0.588, *P* = 0.035 for raffinose and R = − 0.555, *P* = 0.040 for stachyose) was also observed. However, whether the decreased levels of oligosaccharides in serum from neonatal chicks is related to the enrichment of SCFAs-producing bacteria involving in modulation of glycometabolism of host [59], which needs to be explored further.

Furthermore, increased taurine levels were found in the Arg injection group. Taurine was reported to exert the protective effects on intestinal damage through its various physiological functions such as antioxidant properties [60–62], while bile acids conjugated by taurine are detrimental to the intestinal mucosal structure of chickens [63]. Besides resulting from abundant Ruminococcaceae promoting 7α-dehydroxylation reaction for generation of secondary bile acids [42], the lower levels of taurine-conjugated bile acids might also be related to the enzyme system catalyzing conjugation in the liver [64]. Thus, the reduction in taurine-conjugated bile acids, including taurochenodeoxycholate, taurodeoxycholate, and tauroursodeoxycholic acid, implies that IOF of Arg may modulate the metabolism of taurine-conjugated bile acids, which favor the intestinal development.

## Conclusions

Prenatal Arg supplementation promoted early intestinal development of post-hatch layer chicks, contributing to the subsequent improvement in growth performance. The metabolic pathways, including enhanced uptake of residual yolk lipid, modulated metabolisms of galactose and taurine-conjugated bile acids, suggest more energy and nutrients could be directed towards rapid growth of intestine at the beginning of post-hatch when embryos received IOF. Simultaneously, the shift of cecal microbiome composition and the colonization of potential probiotics were accelerated by IOF of Arg, which may contribute to modulate host metabolism and improve intestinal development. Early microbial colonization during the first few days may be the key period for the prenatal Arg supplementation to exert these positive effects in layer chicks at early stage. These findings provide new insight into the role of IOF of Arg in the establishment of the gut microbiota of newly-hatched layer chicks, and can expand our fundamental knowledge about prenatal nutrition, early bacterial colonization and intestinal development in neonate.

## Supplementary information

**Additional file 1: Table S1.** Composition and nutrient levels of experimental diets (air-dry basis, %). **Table S2.** Analysis of similarities (ANOSIM) of weighted and unweighted uniFrac distances. **Figure S1.** Intestinal morphological structure in layer chicks on d 3, 14 and 42. **Figure S2.** Differential species identified from cecal microbiota of layer chicks from different groups. **Figure S3.** PCA and PLS-DA analysis base on LC-MS/MS of serum on d 3 and 42.

## Data Availability

The raw data by pyrosequencing of 16S rRNA genes were subjected to Sequence Read Archive (SRA) database (Accession Number: PRJNA564927).

## Abbreviations

ADFI: Average daily feed intake
ADG: Average daily gain
Arg: Arginine
BW: Body weight
CD: Crypt depth
F/G: Feed: Gain
GIT: Gastrointestinal tract
IOF: *in ovo* feeding
LDA: Linear discriminant analysis
LEfSe: Linear discriminant analysis effect size
NO: Nitric oxide
OTUs: Operational taxonomic units
PCA: Principal-component analysis
PCoA: Principal coordinate analysis
PLS-DA: Partial least squares discriminant analysis
SCFAs: Short-chain fatty acids
VH: Villus height

## References

[1] Wu G, Bazer FW, Cudd TA, Meininger CJ, Spencer TE (2004). Maternal nutrition and fetal development. J Nutr.

[2] Stern CD (2005). The chick: a great model system becomes even greater. Dev Cell.

[3] Iji P, Saki A, Tivey D (2001). Body and intestinal growth of broiler chicks on a commercial starter diet. 1. Intestinal weight and mucosal development. Br Poult Sci.

[4] Milani C, Duranti S, Bottacini F, Casey E, Turroni F, Mahony J (2017). The first microbial colonizers of the human gut: composition, activities, and health implications of the infant gut microbiota. Microbiol Mol Biol Rev.

[5] Lan Y, Verstegen M, Tamminga S, Williams B (2005). The role of the commensal gut microbial community in broiler chickens. World’s Poult Sci J.

[6] Rubio LA (2018). Possibilities of early life programming in broiler chickens via intestinal microbiota modulation. Poult Sci.

[7] Noy Y, Geyra A, Sklan D (2001). The effect of early feeding on growth and small intestinal development in the posthatch poult. Poult Sci.

[8] Li S, Zhi L, Liu Y, Shen J, Liu L, Yao J (2016). Effect of *in ovo* feeding of folic acid on the folate metabolism, immune function and epigenetic modification of immune effector molecules of broiler. Br Poult Sci.

[9] Batal A, Parsons CM (2002). Effect of fasting versus feeding oasis after hatching on nutrient utilization in chicks. Poult Sci.

[10] Wu G, Bazer FW, Davis TA, Kim SW, Li P, Rhoads JM (2009). Arginine metabolism and nutrition in growth, health and disease. Amino Acids.

[11] Wu G, Bazer FW, Satterfield MC, Li X, Wang X, Johnson GA (2013). Impacts of arginine nutrition on embryonic and fetal development in mammals. Amino Acids.

[12] Yuan C, Ding Y, He Q, Azzam M, Lu J, Zou X (2015). L-arginine upregulates the gene expression of target of rapamycin signaling pathway and stimulates protein synthesis in chicken intestinal epithelial cells. Poult Sci.

[13] Gao T, Zhao M, Li Y, Zhang L, Li J, Yu L (2018). Effects of *in ovo* feeding of L-arginine on the development of digestive organs, intestinal function and post-hatch performance of broiler embryos and hatchlings. J Anim Physiol Anim Nutr.

[14] Jha R, Singh AK, Yadav S, Berrocoso JFD, Mishra B (2019). Early nutrition programming (*in ovo* and post-hatch feeding) as a strategy to modulate gut health of poultry. Front Vet Sci..

[15] Gao T, Zhao M, Zhang L, Li J, Yu L, Lv P (2017). Effects of *in ovo* feeding of l-arginine on the development of lymphoid organs and small intestinal immune barrier function in posthatch broilers. Anim Feed Sci Technol.

[16] Uni Z, Noy Y, Sklan D (1996). Development of the small intestine in heavy and light strain chicks before and after hatching. Br Poult Sci.

[17] Uni Z, Perry GC (2006). Early development of small intestinal function. Avian gut function in health and disease.

[18] Wilson K, Rodrigues D, Briggs W, Duff A, Chasser K, Bielke L (2019). Evaluation of the impact of in ovo administered bacteria on microbiome of chicks through 10 days of age. Poult Sci.

[19] Roto SM, Kwon YM, Ricke SC (2016). Applications of in ovo technique for the optimal development of the gastrointestinal tract and the potential influence on the establishment of its microbiome in poultry. Front Vet Sci.

[20] Siwek M, Slawinska A, Stadnicka K, Bogucka J, Dunislawska A, Bednarczyk M (2018). Prebiotics and synbiotics–in ovo delivery for improved lifespan condition in chicken. BMC Vet Res.

[21] Edgar RC (2010). Search and clustering orders of magnitude faster than BLAST. Bioinformatics..

[22] Tanja M, Salzberg SL (2011). FLASH: fast length adjustment of short reads to improve genome assemblies. Bioinformatics..

[23] Edgar RC, Haas BJ, Clemente JC, Christopher Q, Rob K (2011). UCHIME improves sensitivity and speed of chimera detection. Bioinformatics..

[24] Edgar RC (2013). UPARSE: highly accurate OTU sequences from microbial amplicon reads. Nat Methods.

[25] Warton DI, Wright ST, Wang Y (2012). Distance-based multivariate analyses confound location and dispersion effects. Methods Ecol Evol.

[26] Qu W, Nie C, Zhao J, Ou X, Zhang Y, Yang S (2018). Microbiome–metabolomics analysis of the impacts of long-term dietary advanced-Glycation-end-product consumption on C57BL/6 mouse fecal microbiota and metabolites. J Agric Food Chem.

[27] Tahmasebi S, Toghyani M (2016). Effect of arginine and threonine administered *in ovo* on digestive organ developments and subsequent growth performance of broiler chickens. J Anim Physiol Anim Nutr.

[28] Gao T, Zhao M, Zhang L, Li J, Yu L, Lv P (2017). Effect of *in ovo* feeding of L-arginine on the hatchability, growth performance, gastrointestinal hormones, and jejunal digestive and absorptive capacity of posthatch broilers. J Anim Sci.

[29] Foye O, Ferket P, Uni Z (2007). The effects of *in ovo* feeding arginine, β-hydroxy-β-methyl-butyrate, and protein on jejunal digestive and absorptive activity in embryonic and neonatal Turkey poults. Poult Sci.

[30] Montagne L, Crévieu-Gabriel I, Toullec R, Lallès J (2003). Influence of dietary protein level and source on the course of protein digestion along the small intestine of the veal calf. J Dairy Sci.

[31] Thum C, Cookson AL, Otter DE, McNabb WC, Hodgkinson AJ, Dyer J (2012). Can nutritional modulation of maternal intestinal microbiota influence the development of the infant gastrointestinal tract?. J Nutr.

[32] Ballou AL, Ali RA, Mendoza MA, Ellis J, Hassan HM, Croom W (2016). Development of the chick microbiome: how early exposure influences future microbial diversity. Front Vet Sci..

[33] Apajalahti J, Vienola K (2016). Interaction between chicken intestinal microbiota and protein digestion. Anim Feed Sci Technol.

[34] Videnska P, Sedlar K, Lukac M, Faldynova M, Gerzova L, Cejkova D (2014). Succession and replacement of bacterial populations in the caecum of egg laying hens over their whole life. PLoS One.

[35] Ding J, Dai R, Yang L, He C, Xu K, Liu S (2017). Inheritance and establishment of gut microbiota in chickens. Front Microbiol.

[36] Duncan SH, Louis P, Flint HJ (2004). Lactate-utilizing bacteria, isolated from human feces, that produce butyrate as a major fermentation product. Appl Environ Microbiol.

[37] Sun Xiaoming, Shen Jinglin, Liu Chang, Li Sheng, Peng Yanxia, Chen Chengzhen, Yuan Bao, Gao Yan, Meng Xianmei, Jiang Hao, Zhang Jiabao (2020). L-arginine and N-carbamoylglutamic acid supplementation enhance young rabbit growth and immunity by regulating intestinal microbial community. Asian-Australasian Journal of Animal Sciences.

[38] Kabir S (2009). The role of probiotics in the poultry industry. Int J Mol Sci.

[39] Mao X, Gu C, Hu H, Tang J, Chen D, Yu B (2016). Dietary Lactobacillus rhamnosus GG supplementation improves the mucosal barrier function in the intestine of weaned piglets challenged by porcine rotavirus. PLoS One.

[40] Grond K, Lanctot RB, Jumpponen A, Sandercock BK (2017). Recruitment and establishment of the gut microbiome in arctic shorebirds. FEMS Microbiol Ecol.

[41] Zhang L, Wu W, Lee YK, Xie J, Zhang H (2018). Spatial heterogeneity and co-occurrence of mucosal and luminal microbiome across swine intestinal tract. Front Microbiol.

[42] Vital M, Rud T, Rath S, Pieper DH, Schlüter D (2019). Diversity of bacteria exhibiting bile acid-inducible 7α-dehydroxylation genes in the human gut. Comput Struct Biotechnol J.

[43] Biddle A, Stewart L, Blanchard J, Leschine S (2013). Untangling the genetic basis of fibrolytic specialization by Lachnospiraceae and Ruminococcaceae in diverse gut communities. Diversity..

[44] Kien CL, Blauwiekel R, Bunn JY, Jetton TL, Frankel WL, Holst JJ (2007). Cecal infusion of butyrate increases intestinal cell proliferation in piglets. J Nutr.

[45] Torok VA, Ophel-Keller K, Loo M, Hughes RJ (2008). Application of methods for identifying broiler chicken gut bacterial species linked with increased energy metabolism. Appl Environ Microbiol.

[46] Diao H, Jiao A, Yu B, Mao X, Chen D (2019). Gastric infusion of short-chain fatty acids can improve intestinal barrier function in weaned piglets. Genes Nutr.

[47] Adamberg K, Adamberg S, Ernits K, Larionova A, Voor T, Jaagura M (2018). Composition and metabolism of fecal microbiota from normal and overweight children are differentially affected by melibiose, raffinose and raffinose-derived fructans. Anaerobe..

[48] De Filippo C, Cavalieri D, Di Paola M, Ramazzotti M, Poullet JB, Massart S (2010). Impact of diet in shaping gut microbiota revealed by a comparative study in children from Europe and rural Africa. Proc Natl Acad Sci.

[49] Song X, Zhong L, Lyu N, Liu F, Li B, Hao Y (2019). Inulin can alleviate metabolism disorders in ob/ob mice by partially restoring leptin-related pathways mediated by gut microbiota. Genom Proteom Bioinf.

[50] Dinh DM, Volpe GE, Duffalo C, Bhalchandra S, Tai AK, Kane AV (2014). Intestinal microbiota, microbial translocation, and systemic inflammation in chronic HIV infection. J Infect Dis.

[51] Lin DS, Connor WE, Anderson GJ (1991). The incorporation of n-3 and n-6 essential fatty acids into the chick embryo from egg yolks having vastly different fatty acid compositions. Pediatr Res.

[52] Lilburn M (1998). Practical aspects of early nutrition for poultry. J Appl Poult Res.

[53] Noy Y, Sklan D (2001). Yolk and exogenous feed utilization in the posthatch chick. Poult Sci.

[54] Noble R, Shand J (1985). Unsaturated fatty acids compositional changes and desaturation during the embryonic development of the chicken (Gallus domesticus). Lipids..

[55] Noble R, Ogunyemi D (1989). Lipid changes in the residual yolk and liver of the chick immediately after hatching. Neonatology..

[56] Yu L, Gao T, Zhao M, Lv P, Zhang L, Li J (2017). *In ovo* feeding of *L*-arginine alters energy metabolism in post-hatch broilers. Poult Sci.

[57] Brady LJ, Romsos DR, Leveille GA (1979). Gluconeogenesis in isolated chicken (Gallus domesticus) liver cells. Comp Biochem Phys B.

[58] Kuhre RE, Ghiasi SM, Adriaenssens AE, Albrechtsen NJW, Andersen DB, Aivazidis A (2019). No direct effect of SGLT2 activity on glucagon secretion. Diabetologia..

[59] Hur KY, Lee MS (2015). Gut microbiota and metabolic disorders. Diabetes Metab J.

[60] Son M, Kim HK, Kim WB, Yang J, Kim BK (1996). Protective effect of taurine on indomethacin-induced gastric mucosal injury. Arch Pharm Res.

[61] Son MW, Ko JI, Doh HM, Kim WB, Park TS, Shim MJ (1998). Protective effect of taurine on TNBS-induced inflammatory bowel disease in rats. Arch Pharm Res.

[62] Tsuchioka T, Fujiwara T, Sunagawa M (2006). Effects of glutamic acid and taurine on total parenteral nutrition. J Pediatr Surg.

[63] Huang C, Guo Y, Yuan J (2014). Dietary taurine impairs intestinal growth and mucosal structure of broiler chickens by increasing toxic bile acid concentrations in the intestine. Poult Sci.

[64] Kase B, Björkhem I (1989). Peroxisomal bile acid-CoA: amino-acid N-acyltransferase in rat liver. J Biol Chem.

