# Supplementary material for: Dynamic alterations in early intestinal development, microbiota and metabolome induced by in ovo feeding of L-arginine in a layer chick model
Source: J Anim Sci Biotechnol. 2020 Mar 10;11:19. doi: 10.1186/s40104-020-0427-5 (PMC7063725; doi:10.1186/s40104-020-0427-5)
Supplement: Supplementary file 1 — Additional file 1: Table S1. Composition and nutrient levels of experimental diets (air-dry basis, %). Table S2. Analysis of similarities (ANOSIM) of weighted and unweighted uniFrac distances. Figure S1. Intestinal morphological structure in layer chicks on d 3, 14 and 42. Figure S2. Differential species identified from cecal microbiota of layer chicks from different groups. Figure S3. PCA and PLS-DA analysis base on LC-MS/MS of serum on d 3 and 42. [file 40104_2020_427_MOESM1_ESM.docx]

**Table S1.** Composition and nutrient levels of experimental diets (air-dry basis, %).

| Items | Value | Nutrient levels | Value |
| --- | --- | --- | --- |
| Ingredient |  | Calculated nutrient level^2^ |  |
| Corn | 66.00 | AME (MJ/kg) | 12.02 |
| Soybean meal | 26.30 | Crude protein | 19.44 |
| Cottonseed protein | 2.00 | Calcium | 0.95 |
| Wheat | 1.50 | Total phosphorus | 0.65 |
| Dicalcium phosphate | 1.70 | Available phosphorus | 0.41 |
| Limestone | 1.39 | Lysine | 1.04 |
| Salt | 0.10 | Methionine + cystine | 0.77 |
| Sodium bicarbonate | 0.10 | Threonine | 0.71 |
| Sodium sulfate | 0.27 | Tryptophan | 0.24 |
| Choline chloride | 0.10 |  |  |
| L-Lysine HCl | 0.20 |  |  |
| DL-methionine | 0.20 |  |  |
| Premix^1^ | 0.14 |  |  |
| Total | 100.00 |  |  |

^1^The premix provided the following per kg of diets: VA, 12500 IU, VD_3_, 4125 IU, VE 15 IU, VK, 2 mg, VB_6_, 6 mg, VB_12_, 0.08 mg, niacin 32.5 mg, riboflavin 8.5 mg, thiamine, 2.5 mg, calcium pantothenate, 50 mg, biotin, 2 mg, pyridoxine, 8 mg, folic acid, 2.5 mg, choline, 500 mg, Se, 0.3 mg, Mn, 65 mg, Fe 60 mg, Cu 15 mg, I 1 mg, Zn, 66 mg, phytase, 500 mg. ^2^Nutrient levels were calculated values.

**Table S2.** Analysis of similarities (ANOSIM) of weighted and unweighted uniFrac distances.

| Sampling time | Unweighted UniFrac distance | | Weighted UniFrac distance | |
| --- | --- | --- | --- | --- |
|  | R-value | *P*-value | R-value | *P*-value |
| d 3 | 0.607 | 0.001 | 0.292 | 0.004 |
| d 14 | 0.210 | 0.001 | 0.061 | 0.079 |
| d 42 | 0.021 | 0.345 | 0.053 | 0.185 |


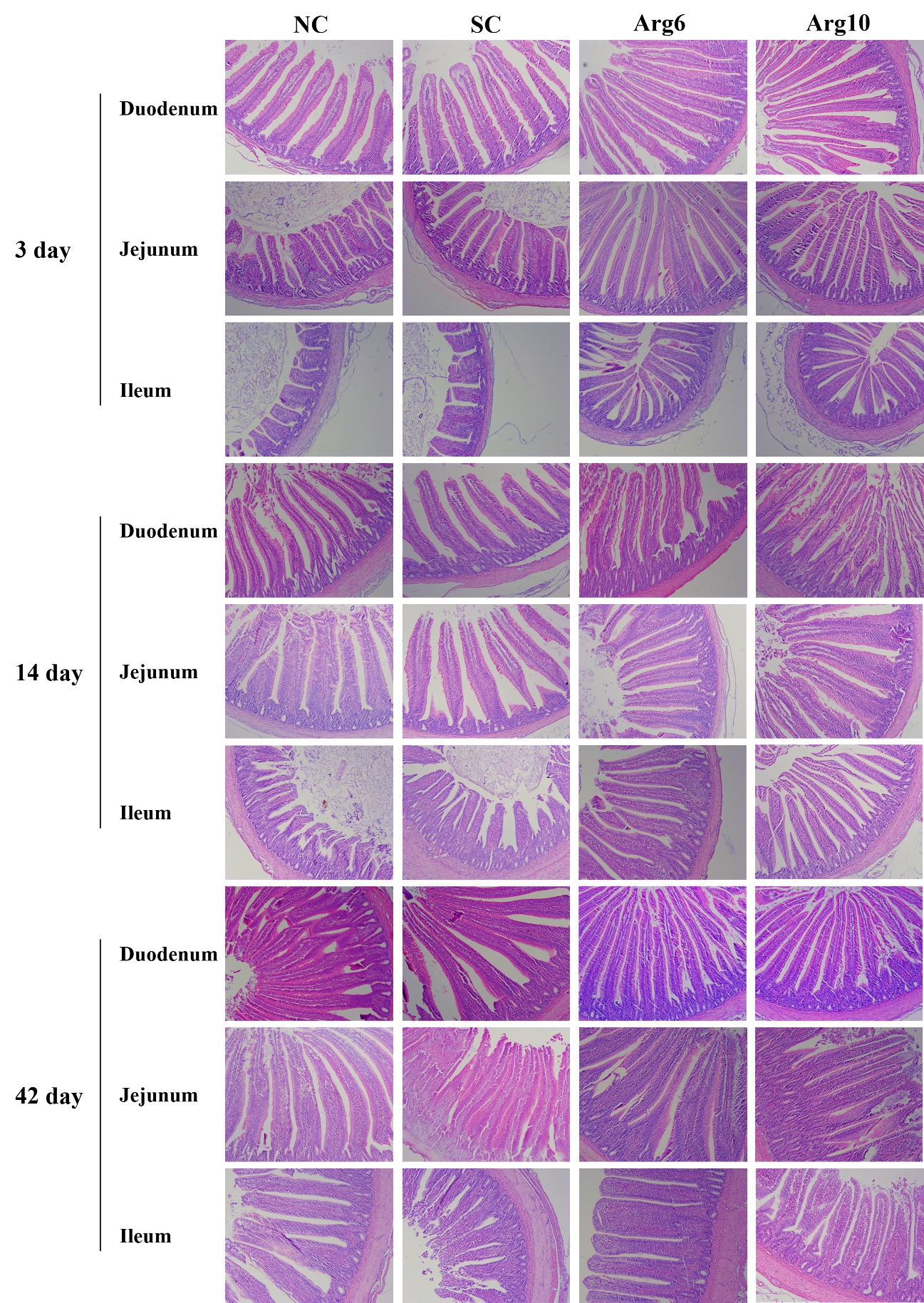


**Fig. S1** Intestinal morphological structure in layer chicks on d 3, 14 and 42. The pictures were observed at 100× magnification (n=8). NC, non-injected control group; SC, saline (0.85% NaCl)-injected control group; Arg6, injected with 6 mg Arg; Arg10, injected with 10 mg Arg.


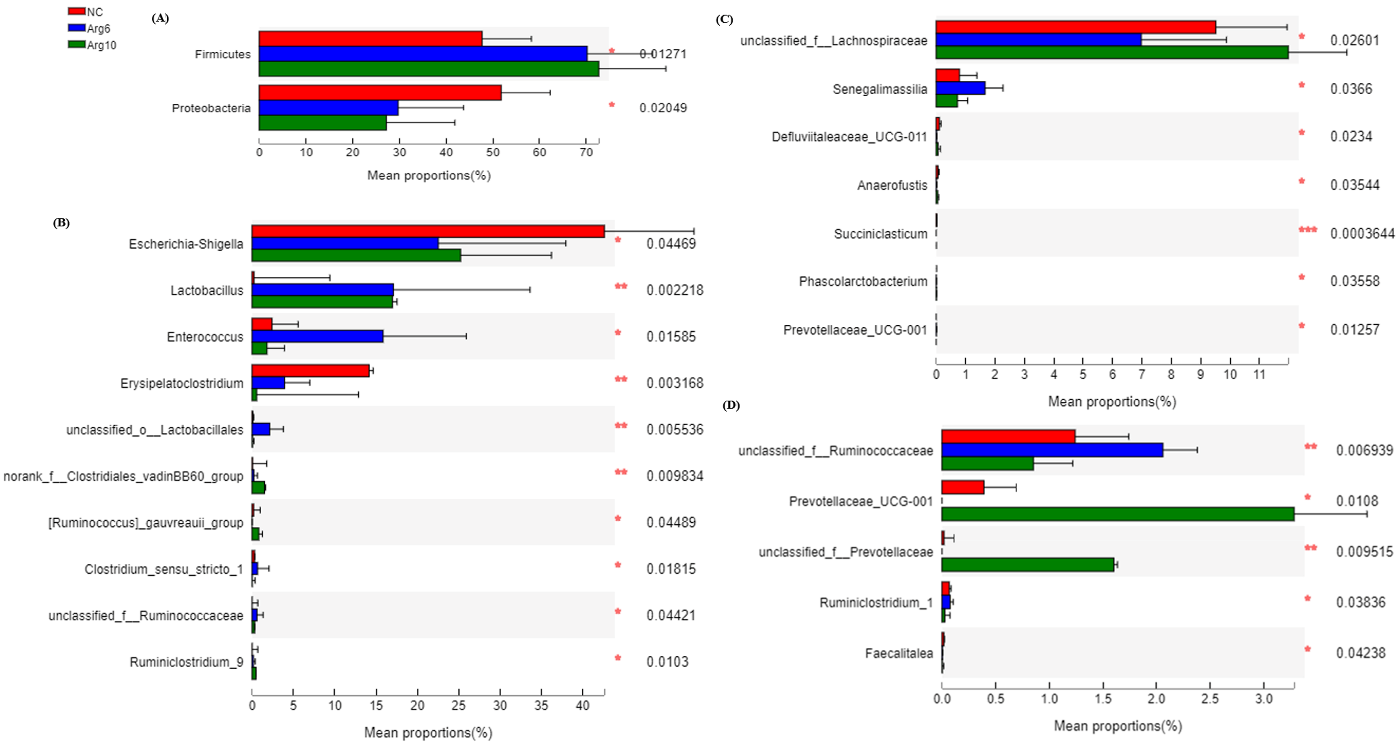


**Fig. S2** Differential species identified from cecal microbiota of layer chicks from different groups. A and B were differential species identified at phylum and genus level on d 3 respectively. C and D were differential species identified at genus level on d 14 and 42 respectively (No differential species identified at phylum level).


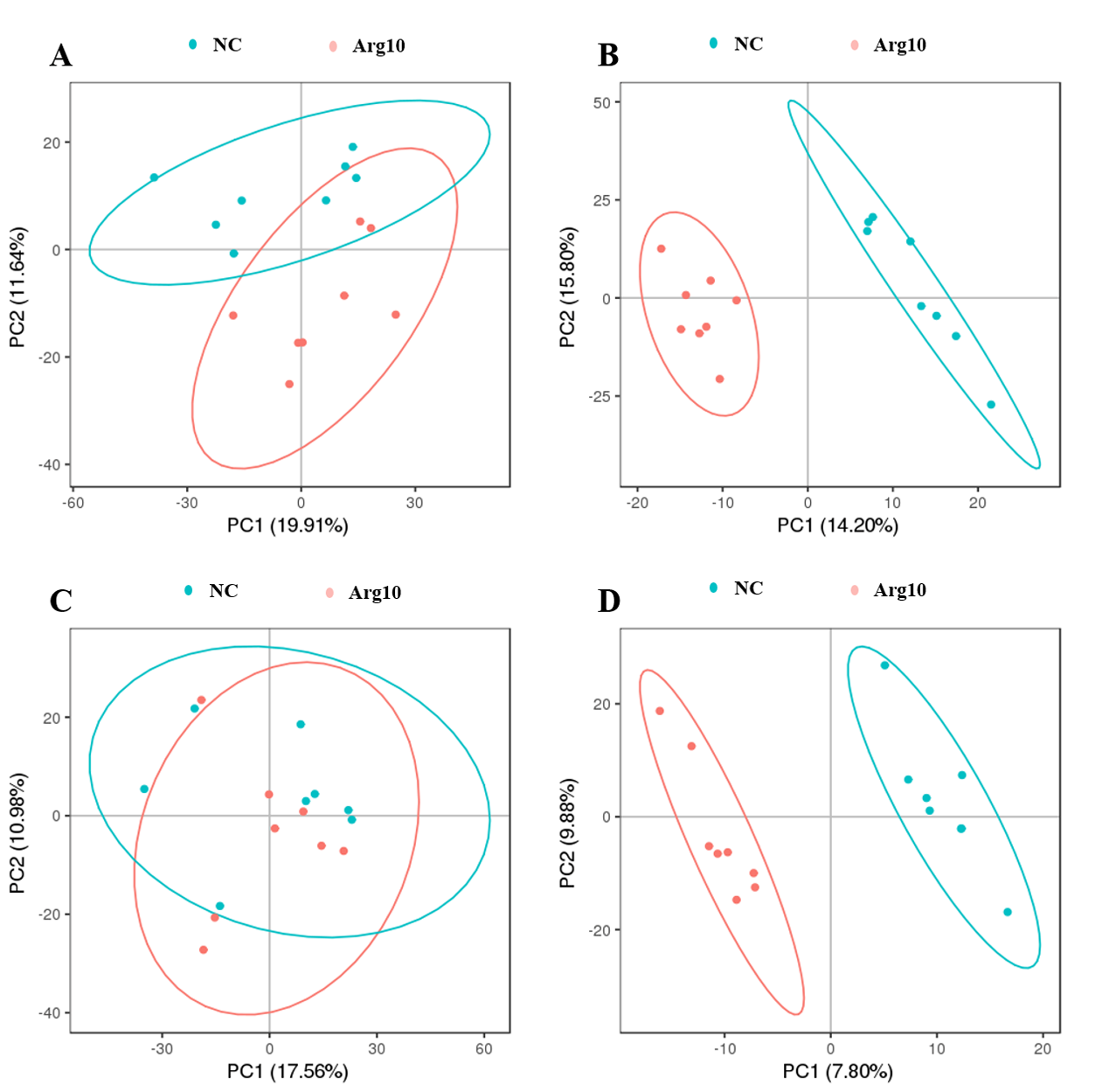


**Fig. S3** PCA and PLS-DA analysis base on LC-MS/MS of serum on d 3 and 42. A, PCA score plots on d 3; B, PLS-DA score plots on d 3; C, PCA score plots on d 42; D, PLS-DA score plots on d 42. Each data point represents one bird serum sample (n=8). NC, non-injected control group; Arg10, injected with 10 mg Arg.
